# Insights into Adsorption Behaviors of Multi-Component Shale Oil in Illite Nanopores Under Different Reservoir Conditions by Molecular Simulation

**DOI:** 10.3390/nano15030235

**Published:** 2025-02-03

**Authors:** Lingtan Zhang, Maojin Tan, Xuefeng Liu, Xiaoqing Lu, Qian Wang, Siyu Wang, Min Tian, Junjie Wang

**Affiliations:** 1 School of Geophysics and Information Technology, China University of Geosciences, Beijing 100083, China; zhanglt@email.cugb.edu.cn (L.Z.); wang_sy@email.cugb.edu.cn (S.W.); 2 College of Science, China University of Petroleum (East China), Qingdao 266580, China; liuxf@upc.edu.cn; 3 School of Materials Science and Engineering, China University of Petroleum (East China), Qingdao 266580, China; luxq@upc.edu.cn; 4 Sinopec Geophysical Research Institute Co., Ltd., Nanjing 211103, China; wangq.swty@sinopec.com; 5 Research Institute of Exploration and Development, Sinopec Oilfield Company, Dongying 257015, China; tianmin502.slyt@sinopec.com; 6 Tuha Division, China Petroleum Logging Co., Ltd., Hami 839009, China; wjjthcj@cnpc.com.cn

**Keywords:** illite nanopore, adsorption behavior, multi-component shale oil, molecular dynamics simulation

## Abstract

Clay pores are important storage spaces in shale oil reservoirs. Studying the adsorption behavior of shale oil in clay nanopores is of great significance for reserve assessment and exploitation. In this work, illite clay pore models and multi-component shale oil adsorption models considering light hydrocarbon correction are constructed for carrying out molecular dynamics simulation. We studied the adsorption behavior and characteristics of shale oil in illite pores, and analyzed the effects of reservoir environmental factors such as temperature, pressure and pore size on the adsorption behavior. The results show that in illite nanopores, shale oil can form multiple adsorption layers. The heavier the component, the stronger the interaction with the wall. The adsorption ratio of the component is closely related to the solid–liquid interaction and the molar fraction, which preliminarily reveals the reason why the heavy component content in the produced oil is considerable. The increase in temperature promotes the desorption of light and medium components, while the heavy components and dissolved gas are less affected; although the increase in pressure inhibits diffusion, the adsorption amount changes little, and only the light component increases slightly. This study deeply reveals the adsorption mechanism of shale oil in illite pores, providing a theoretical basis for the optimization and development of shale reservoirs.

## 1. Introduction

As the global demand for oil and gas energy continues to increase, unconventional oil and gas, especially shale oil and gas, has received more and more attention and is changing the global energy system [1,2]. The exploration and development of shale oil and gas has achieved remarkable success in North America, which has enabled the rapid growth of oil production in the United States [3,4,5]. Compared with conventional oil resources, shale oil resources have large reserves and great development potential. It is generally believed that shale oil mainly exists in organic and inorganic nanopores or microcracks in shale, and the storage and migration of shale oil depend on the pore type, pore structure and physical properties of the reservoir [6]. Therefore, a deep understanding and accurate characterization of the adsorption and diffusion behavior of shale oil in nanopores is crucial for shale oil reserve assessment and exploitation.

Shale reservoirs have developed nanopores and extremely low permeability, which poses a major challenge to understanding the adsorption and diffusion behavior of shale oil in nanopores [7]. An accurate understanding of the pore structure is crucial to understanding the adsorption behavior of shale oil [8,9,10]. Previous researchers have studied the pore structure of shale through a large number of microscale experimental methods. Liu et al. [11] and Loucks et al. [12] conducted experimental studies on the pore structure of Bakken shale samples and Barnett shale samples in the United States by optical means such as atomic force microscopy (AFM), focused ion beam scanning electron microscopy (FIB-SEM) and field emission scanning electron microscopy (FE-SEM), and found that nanopores were developed in large quantities. Statistical results showed that the pores were mainly concentrated in the mesopore (2–50 nm), and 5–15 nm pores dominated. Chen et al. [13] characterized the pore structure of shale in southern China by FE-SEM, low-temperature N_2_ adsorption, and high-pressure mercury intrusion porosimetry (HMIP), and found that the pore width mainly ranged from 2 nm to 10 nm. Compared with traditional reservoirs, the nano-constraints of a large number of nanopores in shale reservoirs significantly enhance the influence of the pore surface on fluid molecules [14]. As a typical lacustrine sedimentary basin in China, the Songliao Basin has a particularly high content of clay minerals such as illite, montmorillonite and kaolinite, reaching 40–60% of the total minerals [15]. The clay minerals in the shale of the Qingshankou Formation Qijia-Gulong Sag are highly evolved, with a large amount of montmorillonite converted into illite and a large amount of silica precipitated. The shale is more brittle and has good compressibility. It is the main shale oil producing area in the Songliao Basin. Recent studies have shown that the large number of nanopores developed in shale clay minerals constitute an important reservoir space [16], and the clay minerals show significant adsorption capacity for metal ions, protons and organic molecules [17,18].

For the study of the adsorption behavior of shale oil in nanopores, traditional experimental methods mainly include isothermal adsorption, nuclear magnetic resonance (NMR), X-ray diffraction (XRD), scanning electron microscopy (SEM) analysis and hydrocarbon diffusion experiments [8,10,19,20]. It also includes Soxhlet extraction or Rock-Eval pyrolysis to separate shale oil from the rock surface to obtain reservoir information [19,21,22,23]. Although the above methods provide reliable experimental data for the study of shale oil adsorption characteristics to a certain extent, the experimental methods are still difficult to cope with under the conditions of complex shale reservoir environments such as high temperature, high pressure and multi-component oil and gas. They can only obtain macroscopic adsorption characteristics, but cannot provide more detailed microscopic adsorption behavior, making it difficult to explain the mechanism [24]. Molecular dynamics (MD) simulation is an effective method to meet these challenges. Based on a mature potential energy model, MD simulation can obtain results that are consistent with experiments without introducing too many assumptions. It can effectively deal with nanoscale adsorption and diffusion behaviors, provide micromechanical explanations, and facilitate the realization of reservoir environmental conditions such as high temperature and high pressure that are difficult to achieve in experiments [25]. A large number of studies have shown that MD simulation is a reliable method for studying the behavior of hydrocarbons in nano-confined spaces [26,27,28,29,30,31,32]. Wang et al. [33] analyzed the static structural characteristics of octane in quartz nanopores based on equilibrium molecular dynamics (EMD) simulation and found that stratification occurred at the solid–liquid interface and octane existed in a “solid-like” form. Cao et al. [34] studied the adsorption and diffusion behavior of liquid hydrocarbons on the surfaces of various minerals through MD simulation and found that the type of hydrocarbon molecules and the temperature and pressure environment of the reservoir significantly affected the adsorption characteristics. Zhang et al. [35] emphasized the impact of nanoscale effects on gas storage and transmission, and pointed out the importance of simulating multi-component hydrocarbons and complex pore networks. They found that the adsorption capacity and diffusion of supercritical methane were significantly affected by pressure, mineral type and water content. Yang et al. [26] used MD simulation to study the adsorption behavior of multi-component shale oil in kerogen fractures and analyzed the effects of temperature and pore size on adsorption behavior. Fang et al. [36] established a kerogen-illite heterogeneous wall pore model and used MD to simulate the composite wall stacking effect on the occurrence of shale oil. The effects of temperature, pore size and wall component ratio on the adsorption ratio and diffusion capacity of shale oil were also studied. Wang et al. [37,38] studied the fluid flow in the fractured porous media produced by hydraulic fracturing and gave the mathematical formula for two-phase flow in fractured porous media, finding that the adsorption behavior of shale oil also changes the flow pattern and efficiency of shale oil. Zhan et al. [39] used MD simulation to study the adsorption and flow of n-alkanes in graphene channels and real kerogen slits with different surface roughness. They found that it is necessary to consider the atomic roughness to obtain acceptable results when using graphene channel for simplicity. Zhang et al. [40] used MD simulation to investigate adsorption behaviors and mass transfer of CO_2_ and n-decane molecules in multiple quartz nanopores. They found that as the pore width decreased, the surface adsorption of n-decane was significantly weakened, but that of CO_2_ was enhanced, and the maximum oil recovery rate could be achieved.

The above studies have enriched the understanding of the adsorption characteristics of hydrocarbons in shale and provided many valuable insights. However, it should be pointed out that shale fluids in the currently reported studies are mostly modeled based on single components or produced oil components, and the loss of light hydrocarbons in shale oil production is rarely considered. In fact, due to the decrease in temperature and pressure during shale production, a large number of light hydrocarbon components contained in formation shale oil, especially medium- and high-maturity shale oil, are seriously lost. The produced oil components cannot fully represent the shale oil components under reservoir conditions. When studying shale oil under reservoir conditions, light hydrocarbon correction must be performed. On the other hand, shale oil is enriched in different types of reservoir spaces in shale reservoirs, and the content of shale clay minerals is very high, but there are few reports on the occurrence state and adsorption behavior of shale oil in clay pores.

In this study, we used the pyrolysis–gas chromatography (PY–GC) experimental data of the pressure-maintained core of the Qingyi Section of Qingshankou Formation in the Gulong Sag, Songliao Basin, China, and constructed a multi-component shale oil molecular model with light hydrocarbon correction for MD simulation. We established illite pores based on the main clay mineral of Gulong shale, and deeply revealed the adsorption characteristics of multi-component shale oil in illite clay pores from a microscopic scale. We also discussed the effects of different reservoir conditions including temperatures, pressures, and pore sizes on adsorption behavior. This study deepened the understanding of the adsorption mechanism of shale oil in clay pores and provided a scientific basis for the evaluation and development of shale oil.

## 2. Illite Nanopore Model Construction

**Figure 1:**
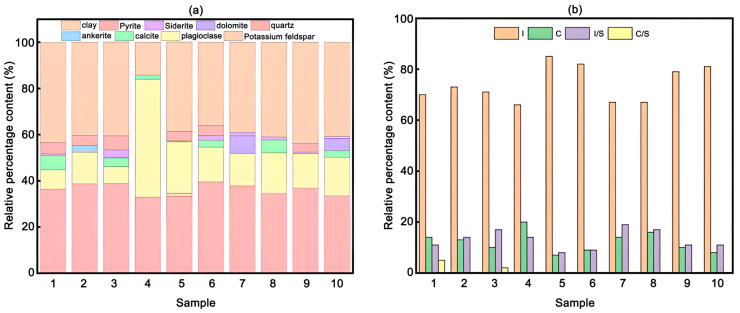
Shale mineral composition. (**a**) Composition of the whole shale; (**b**) Clay mineral composition [41].

Liang et al. [41] used X-ray diffraction (XRD) to analyze the mineral composition of 10 shale samples from the Qingyi section of the Qingshankou Formation in the Songliao Basin. The results showed that the main mineral compositions are clay minerals and quartz, as shown in Figure 1a. The main clay minerals are illite (I), chlorite (C), and illite–montmorillonite mixed-layer minerals (I/S), as shown in Figure 1b. Their average contents are 74.1%, 12.1%, and 13.1%, respectively, and illite also accounts for 83.1% of the illite–montmorillonite mixed-layer minerals. Therefore, illite is the main component of clay minerals [41].

In order to investigate the occurrence characteristics of multi-component shale oil in shale pores, K-illite was selected as an adsorbent in the shale matrix. Illite is a 2:1 clay mineral composed of two layers of silicon–oxygen tetrahedrons sandwiched by a layer of aluminum–oxygen octahedrons. Its initial chemical formula is Si_8_Al_4_O_20_(OH)_4_. Under natural conditions, K-illite will undergo isostructural substitution [42]. Trivalent aluminum atoms (Al^3+^) will replace tetravalent silicon atoms (Si^4+^) in silicon–oxygen tetrahedrons, resulting in electronegativity on the surface. Interlayer potassium ions (K^+^) will balance this electronegativity. The structural formula of the replaced illite is K(Si_7_Al)Al_4_O_20_(OH)_4_. This illite model has been widely used in the study of nanopore adsorption, diffusion performance and hydrodynamic properties [43]. In this study, the illite unit cell is derived from the American Mineralogist Crystal Structure Database (AMCSD); the space group is C2/m, and the unit cell parameters are a = 5.1994, b = 8.9815, c = 10.233, α = 90°, β = 101.6°, γ = 90°. The atomic coordinate parameters are shown in Table 1.

**Figure 2:**
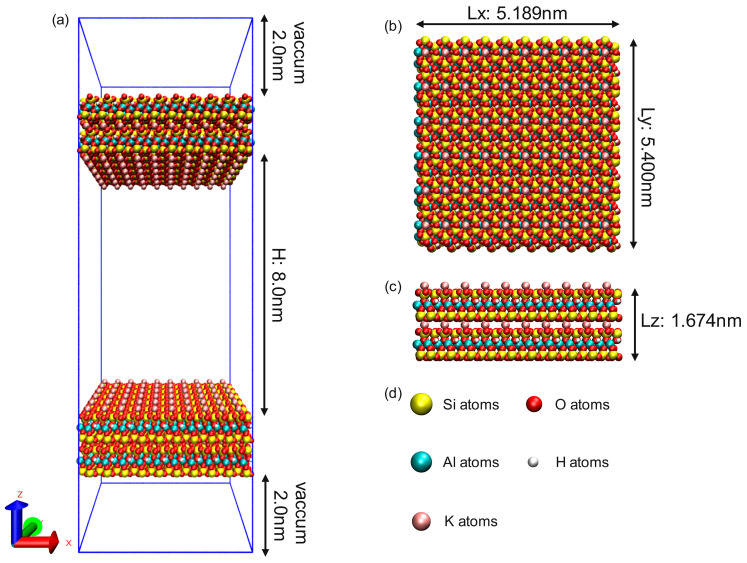
Molecular model of K-illite wall and pores. (**a**) Illite 8.0 nm pores; (**b**) xy plane of illite pores; (**c**) Illite wall; (**d**) Atomic color: yellow Si, red O, green Al, white H, brown K.

The illite pore model constructed in this study contains 120 illite unit cells (10 × 6 × 2) on each wall, and the dimensions of the wall along the x, y, and z directions are 5.189 nm, 5.400 nm, and 1.674 nm, respectively. The illite pore width is defined as the distance between the two oxygen atoms closest to the upper and lower surfaces, and the initial slit aperture is 8.0 nm. The simulation box is set to periodic boundary conditions in the x, y, and z directions. The box contains not only illite walls and pores, but also a 2 nm vacuum layer outside of the solid wall to minimize the periodic charge effect [44,45], as shown in Figure 2.

## 3. Shale Oil Model Construction and Molecular Dynamics Method

### 3.1. Molecular Model of Shale Oil

**Figure 3:**
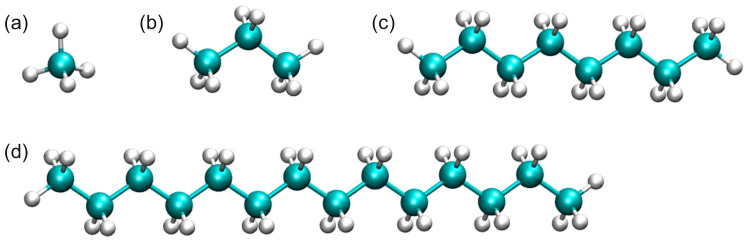
Molecular models of representative components of shale oil (**a**) CH_4_; (**b**) C_3_H_8_; (**c**) C_8_H_18_; (**d**) C_14_H_30_.

The chemical composition of shale oil is very complex, mainly including saturated hydrocarbons, aromatic hydrocarbons, non-hydrocarbons and asphaltene. Different shale oil components have different occurrence characteristics in illite nanopores. Through experimental analysis of the oil produced in the Qingyi section of the Gulong Sag in the Songliao Basin, it is known that saturated hydrocarbons account for 89.42% by mass and are the main material composition of shale oil [46]. The vitrinite reflectance (R_0_) of shale in the Gulong Sag is generally higher than 1.0%. This indicates that the organic matter is in the medium to high maturity stage, liquid hydrocarbons are generated in large quantities, and the oil is relatively light. Therefore, this paper uses multi-component normal alkanes to construct a multi-component shale oil model, with methane (C1), propane (C3), octane (C8) and tetradecane (C14) representing the dissolved gas, light hydrocarbons, medium hydrocarbons and heavy hydrocarbons in shale oil [47,48], respectively, as shown in Figure 3. It should be pointed out that these components are still simplified models compared with the complex components of shale oil obtained in the experiment. In particular, aromatics, non-hydrocarbons, asphaltene and other components are temporarily ignored during modeling due to their low content [26,48,49,50]. Nevertheless, the combination of these components represents the vast majority of shale oil components (about 90%), so they can be used as a research model for shale oil. In addition, there are no alkane components of the same type with particularly close carbon numbers in the alkane system of the model, so the interactions and differences between light, medium and heavy components can be made more obvious, which is conducive to better presenting the occurrence characteristics of different types of shale oil components in the pores and clearly displaying the results.

Based on the pyrolysis–gas chromatography (PY–GC) experimental data of pressure-cored well G1 of the Qingshankou Formation in the Gulong Sag of the Songliao Basin [46], the number of representative components of the multi-component shale oil model was determined, as shown in Table 2. Since the pressure-cored shale samples well retain the gaseous hydrocarbons and some light hydrocarbons that are easily lost, the multi-component shale oil model established in this study effectively recovers the light hydrocarbons, making the model more consistent with the actual reservoir.

**Figure 4:**
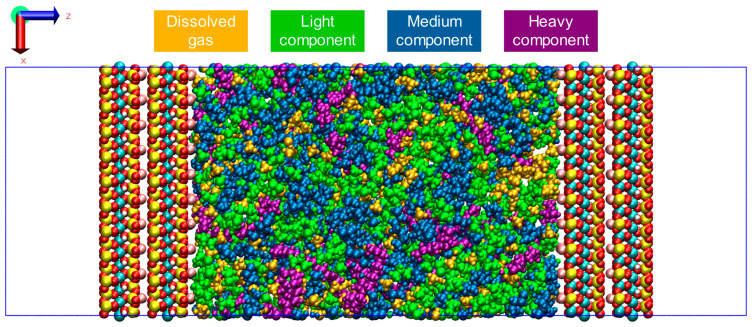
Initial configuration of multi-component shale oil in illite pore. The molecules of different components in the pores are represented by different colors. Brown—Dissolved gas, Green—Light component, Dark blue—Medium component, Purple—Heavy component.

The initial configuration of multi-component shale oil with 8 nm illite pores constructed according to Table 2 is shown in Figure 4.

### 3.2. Force Field and Simulation Method

In this study, we use the Large-scale Atomic/Molecular Massively Parallel Simulator (LAMMPS) software package for all molecular dynamics simulation calculations, and the model and calculation results are visualized by open source software Visual Molecular Dynamics (VMD) [51,52]. Optimized Potentials for Liquid Simulations–All Atoms (OPLS–AA) force field is used to characterize methane and propane molecules. Octane and tetradecane are described by the L–OPLS–AA force field modified by Siu et al. [53], which can more accurately characterize the viscosity, diffusion coefficient and other parameters of long-chain alkanes [53]. Illite minerals and K+ are described by the ClayFF force field [54]. These force fields have been widely used in the study of adsorption, surface wetting and flow behavior of clay nanopores under reservoir conditions. Detailed force field parameters are listed in Table 3, Table 4 and Table 5.

The interactions among alkanes, clay surface and ions are contributed by van der Waals interactions and Coulomb interactions, which are described by the Lennard–Jones (LJ) 12-6 potential function and Coulomb potential as follows, respectively.

(1) $$u\left(r_{ij}\right)=4\varepsilon_{ij}\left[{\left(\frac{\sigma_{ij}}{r_{ij}}\right)}^{12}-{\left(\frac{\sigma_{ij}}{r_{ij}}\right)}^{6}\right]+\frac{q_{i}q_{j}}{4\pi \varepsilon_{0}r_{ij}}$$

in which $r_{ij}$, $\varepsilon_{ij}$ and $\sigma_{ij}$ are the separation distance between atoms, LJ energy and size parameters, respectively; $q_{i}$ and $q_{j}$ represent the partial charges of site *i* and *j*; $\varepsilon_{0}$ is the dielectric constant of vacuum. The Nosé–Hoover thermostat method was used for temperature control and the Berendsen barostat was used for pressure control. The LJ potentials were truncated at 1.0 nm with tail corrections and Coulomb interactions were computed using standard the three-dimensional particle-mesh Ewald method with correction [55,56]. We used the Particle–Particle–Particle–Mesh (PPPM) summation method to calculate the long-range electrostatic interaction. The pairwise interactions between atoms of different types were calculated by the standard Lorentz–Berthelot combining rules as follows [57]:

(2) $$\sigma_{ij}=\frac{1}{2}\left(\sigma_{i}+\sigma_{j}\right)$$

(3) $$\varepsilon_{ij}=\sqrt{\varepsilon_{i}\varepsilon_{j}}$$

where $\sigma_{i}$ and $\sigma_{j}$ are the LJ size parameters of site *i* and *j*; $\varepsilon_{i}$ and $\varepsilon_{j}$ are the LJ energy parameters of site *i* and *j*.

According to the typical geological environment and thermodynamic conditions of the Songliao Basin in China, the initial temperature and the initial pressure of the system were set at 353 K, 20 MPa [58], respectively, and an all-atom EMD simulation was performed. Before the calculation, the conjugate gradient method (CG) was used to minimize the energy of the constructed initial model, thereby optimizing the system, mainly eliminating overlapping and too close molecules. Next, a simulation system under certain temperature and pressure should be obtained. There exist several methods to achieve this. Zhan et al. [56] adopt the piston method. They add an external force on the upper pore wall and keep the bottom one static. The system temperature is controlled by explicitly rescaling fluid velocities, and the target pore width is obtained by adjusting the number of fluid molecules in serials of trial simulation. Wang et al. [48] perform EMD simulations under the NVT ensemble, setting the system temperature at the target temperature, and calculating the pressure of the fluid after equilibrium. When the calculated pressure is close enough to the target pressure by adjusting the number of fluid molecules, the system for simulation is obtained. In this work, we first determined the distance between the upper and lower illite walls and kept the walls static, then a certain number of shale oil molecules were accumulated in the pore. After that, EMD simulations under the NVT ensemble were performed. We calculated the bulk density of the fluid when the system was balanced, and compared it with the bulk density at the target temperature and pressure. Next, a series of trial simulations were conducted to obtain the acceptable bulk density by adjusting the number of shale oil molecules. The EMD simulations were then performed under the NVT ensemble with a simulation time of 8 ns and a step size of 1 fs on this system. The system was balanced in the first 6 ns, and the last 2 ns were used for data collection and statistical analysis.

## 4. Results and Discussion

### 4.1. Force Field Verification

**Figure 5:**
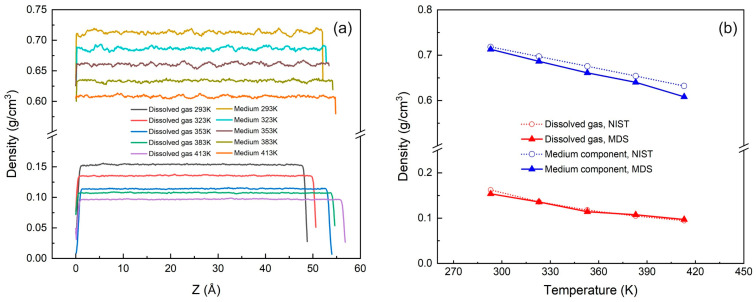
(**a**) Density distribution of methane and octane at 20 MPa at different temperatures (293 K, 323 K, 353 K, 383 K, 413 K); (**b**) Comparison of molecular dynamics simulation of methane and octane density with NIST experimental data.

Before the simulation started, the selected force field was first verified to ensure the correctness of the simulation parameters and the credibility of the simulation results. To this end, we carried out molecular dynamics simulation on the fluid state of methane and octane at 20 MPa pressure and temperatures of 293 K, 323 K, 353 K, 383 K, and 413 K, respectively. There are 743 methane molecules and 599 octane molecules in the model. The initial sizes of the simulation box in the x, y, and z directions are 5.189 nm, 5.400 nm, and 4.996 nm, respectively, and the initial densities are 0.1414 g/cm^3^ and 0.8106 g/cm^3^, respectively. The force field parameters in Table 3, Table 4 and Table 5 were used to perform simulation under the NPT ensemble. The system was balanced in the first 6 ns, and the equilibrium data of the last 2 ns were taken for density calculation. The results are shown in Figure 5.

The results show that after 8 ns of NPT ensemble simulation, methane and propane reach a good equilibrium state at different temperatures, and the spatial density distribution is basically uniform, but the spatial density fluctuation of long carbon chain molecules is slightly greater than that of short carbon chain molecules, as shown in Figure 5a; the density simulation values at each temperature are very consistent with the experimental data of the National Institute of Standards and Technology (NIST), as shown in Figure 5b, with an average error of 2.47%. It verifies the accuracy and reliability of the force field parameters used in this study.

### 4.2. Single-Component Adsorption Characteristics of Shale Oil

**Figure 6:**
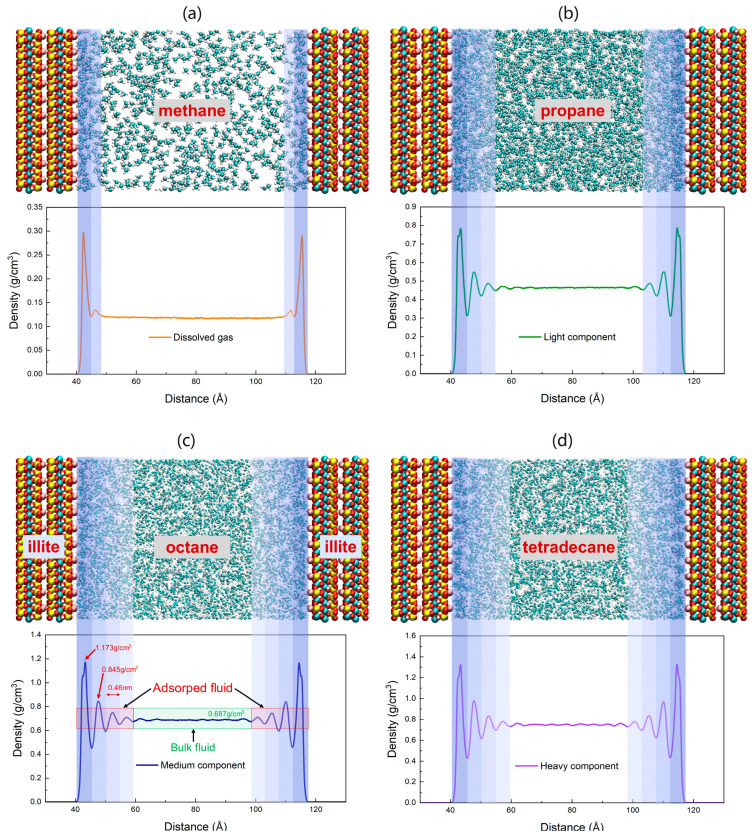
Single component adsorption state and mass density distribution of shale oil in 8 nm illite pores at 353 K and 20 MPa. The blue bars of different depths in subfigures (**a**–**d**) represent the adsorption amount of each component in order to have an intuitive representation. (**a**) methane; (**b**) propane; (**c**) octane; (**d**) tetradecane.

In this work, we first studied the interaction and microscopic behavior of single-component shale oil in illite pores. According to the geological environment of Songliao Basin in China, the temperature was still set to 353 K and the pressure was set to 20 MPa. Figure 6 is a snapshot of the molecular dynamics simulation when each component is in equilibrium under this temperature and pressure condition.

The simulation results show that the distribution of each component molecule in the pore is not uniform, and the mass density shows periodic fluctuation characteristics. Due to the strong solid–liquid interaction near the illite wall, the density curve shows a large amplitude change, and is symmetrically distributed along the pore center on both sides of the wall. As the distance between the component molecules and the wall increases, the effect of the illite wall on the molecules continues to weaken, and the amplitude of the density curve gradually decreases. Near the center of the pore, the molecules show a Brownian motion state, and the density curve is basically non-volatile, which is consistent with the characteristics of free fluid. It is generally believed that the fluctuating part of the density curve corresponds to adsorbed shale oil, and the non-fluctuating part corresponds to free shale oil. Taking octane, a representative substance of the medium component, as an example, the average free density is 0.687 g/cm^3^, which is very consistent with the experimental results (0.676 g/cm^3^) published by NIST, thus verifying the reliability of the simulation results. The peak density of the first adsorption layer close to the illite wall is 1.173 g/cm^3^, which is 1.7 times the density of the free fluid under the same conditions. It can be considered that the adsorption layer exists in a “solid-like” state and is difficult to flow. The peak density of the second adsorption layer is 0.845 g/cm^3^, which is much lower than that of the first adsorption layer. The interaction between the illite wall and octane molecules continues to weaken, and the intermolecular effect gradually becomes the dominant force. Therefore, the density of the third and fourth adsorption peaks is further reduced, and the fluid density gradually transitions to the free fluid density. The thickness of the four adsorption layers is 0.46 nm, which is basically consistent with the width of the normal octane molecule. The octane adsorption accounts for 48.6%, which is close to half. Table 6 gives the detailed parameters of the adsorption layers of other representative components.

The adsorption capacity per unit area $C_{a}$ (${\mathrm{mg}/m}^{2}$) is used to characterize the adsorption capacity of illite and is calculated as follows [58]:

(4) $$C_{a}=\frac{m_{a}}{A_{m}}=\frac{\int_{L_{1}}^{L_{2}}A_{m}\cdot \rho_{m}dL}{A_{m}}$$

In the formula, $A_{m}$ is the illite wall area, $m^{2}$; $m_{a}$ is the mass of adsorbed molecules in the pore, mg; $L_{1}$, $L_{2}$ are the position coordinates of the adsorption area, and $\rho_{m}$ is the mass density of the shale oil component in the pore.

The calculation formula for the adsorption percentage is as follows:

(5) $$P_{a}=\frac{m_{a}+m_{b}}{M}=\frac{\int_{L_{1}}^{L_{2}}A_{m}\cdot \rho_{m}dL+\int_{L_{3}}^{L_{4}}A_{m}\cdot \rho_{m}dL}{\int_{L}A_{m}\cdot \rho_{m}dL}\times 100\%$$

where, $A_{m}$ is the illite wall area, $m^{2}$; $m_{a}$, $m_{b}$ are the masses of molecules adsorbed on the walls on both sides of the pore, mg; $L_{1}$, $L_{2}$, $L_{3}$ and $L_{4}$ are the position coordinates of the adsorption areas on the walls on both sides of the pore, L is the pore width, and $\rho_{m}$ is the mass density of the shale oil component in the pore.

**Figure 7:**
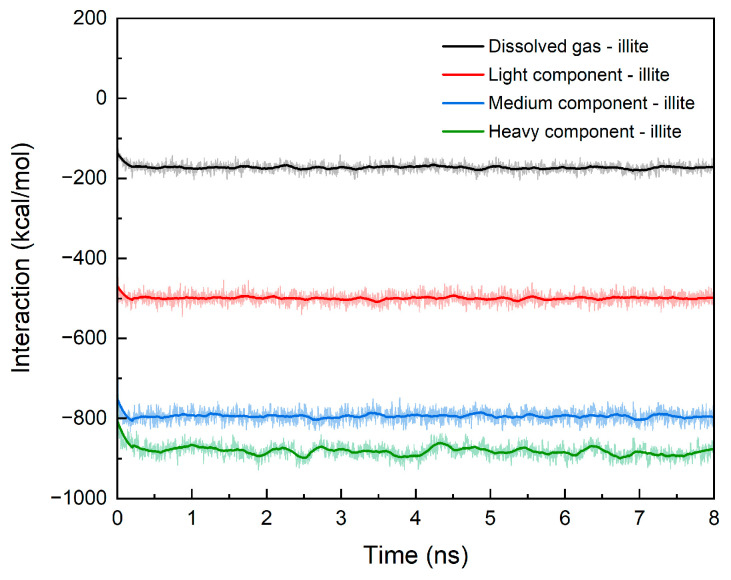
Microscopic interaction energy between different shale oil components and illite wall.

**Figure 8:**
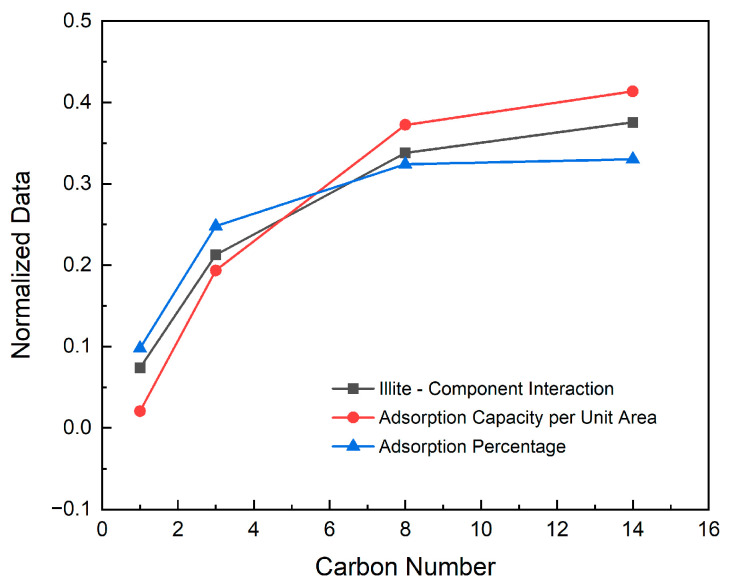
Normalized comparison of solid–liquid interaction energy, adsorption per unit area and adsorption ratio of different shale oil components.

From the adsorption simulation results in Table 6, we found that not only the medium component, but also the average monolayer adsorption thickness of the other three different components in the illite pores is almost the same, and is basically consistent with the width of normal alkanes. The heavy component has more adsorption layers than the light component and medium component, and the total thickness of the adsorption layer is larger. The adsorption of shale oil is caused by interaction between the illite wall and shale oil molecules. In order to reveal the reasons for the different adsorption characteristics, we calculated the interaction energy between the illite wall and each component, as shown in Figure 7. It can be seen that the interaction energy is negative, indicating that there is an attraction between illite and shale oil molecules, and the longer the molecular carbon chain, the greater the attraction, so the number of adsorption layers is also greater. Even when the medium component and the heavy component with the same number of adsorption layers are compared, the free density of the latter also fluctuates slightly. This is the result of the stronger interaction between the illite wall and molecules of the heavy component. Due to the interaction between the wall and the molecules, the small dissolved gas molecules are more likely to gather tightly after adsorption, resulting in the peak density of the dissolved gas adsorption layer being 2.5 times the free density, and the other components are about 1.7 times. The increase in molecular weight corresponds to the increase in the interaction energy between the shale oil component and the wall, resulting in the adsorption capacity per unit area of dissolved gas being less than that of the light component, less than that of the medium component, and less than that of the heavy component. The proportion of the adsorption of each component increases with the increase in carbon chain length. The change trends of the three are compared in Figure 8. For the convenience of comparison, we normalized the data. It can be seen from the figure that the three have the same change trend.

### 4.3. Adsorption Characteristics and Mechanism of Multi-Component Shale Oil

**Figure 9:**
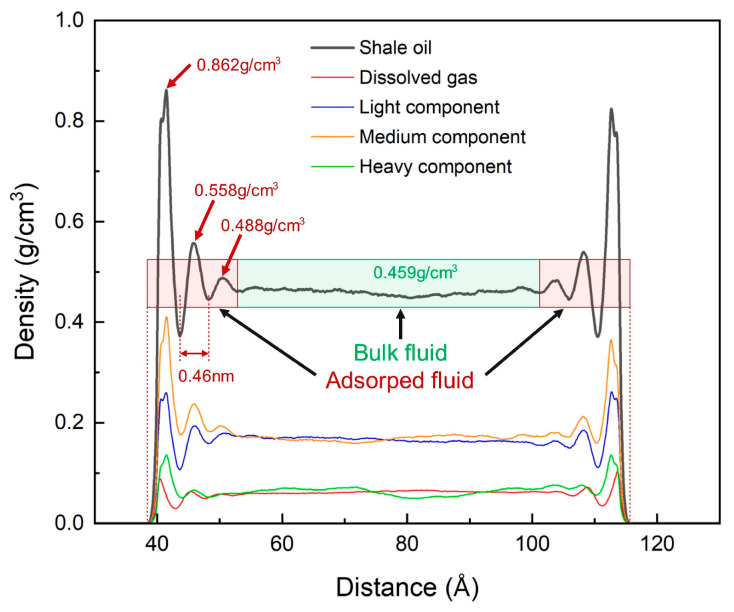
Mass density distribution of shale oil and its components at 353 K and 20 MPa.

We discussed the adsorption behavior and characteristics of a mixture of four representative components (dissolved gas, light component, medium component and heavy component) in shale oil on the pore surface of illite. We conducted EMD simulations at a temperature of 353 K and a pressure of 20 MPa. The density distribution of shale oil and its components is shown in Figure 9. Among the four types of shale oil components, only one representative alkane is selected for each type of component, thereby eliminating the mutual interference of alkanes of different types but with similar carbon numbers on the simulation results, making the mutual influence between the components of shale oil more obvious, and helping to more accurately clarify the adsorption characteristics and mechanisms of multi-component shale oil.

As shown in Figure 9, shale oil forms three adsorption layers on both sides of the illite pores, which are symmetrically distributed along the pore center. The pore center is free shale oil with an average density of 0.459 g/cm^3^, which is very consistent with the density value of 0.451 g/cm^3^ obtained by 8 ns NPT ensemble simulation. Therefore, the simulation results can reveal the adsorption characteristics of mixed alkanes. The adsorption peak densities of the three adsorption layers are 0.862 g/cm^3^, 0.558 g/cm^3^ and 0.488 g/cm^3^, respectively, which are about 1.9 times, 1.2 times and 1.1 times the free state density. The average adsorption layer thickness is 0.46 nm, which is the same as the adsorption layer thickness when each component exists alone. It is approximately the width of the alkane molecule. According to Formula (5), the adsorption amount of shale oil accounts for about 38.3%.

**Figure 10:**
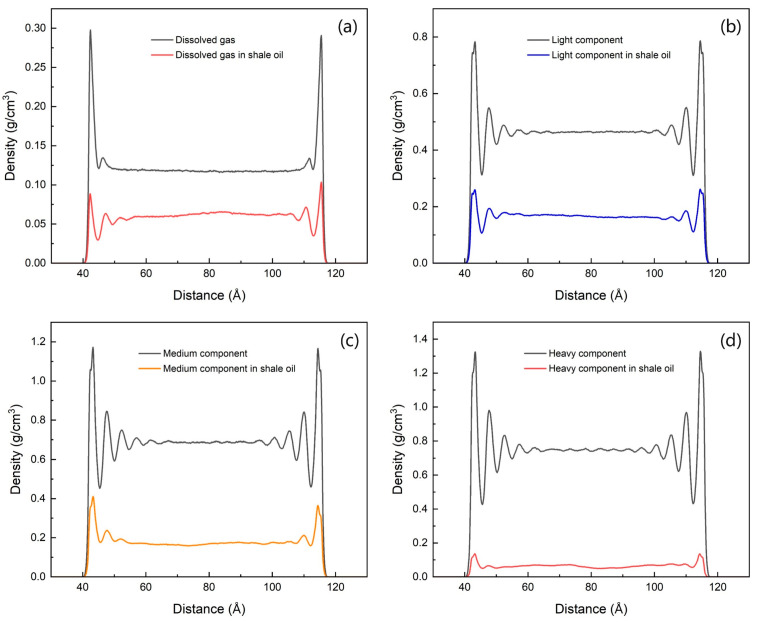
Comparison of density distribution of shale oil components and that of single component. (**a**) dissolved gas (**b**) light component (**c**) medium component (**d**) heavy component.

Due to the existence of competitive adsorption, the density values of the first adsorption peak are in the following order from large to small: medium component, light component, heavy component, and dissolved gas. Although the ratio of the first adsorption peak density to the free state density of the medium component and the heavy component is higher than that when they exist alone, the adsorption ratio is reduced due to the reduction in the number of adsorption layers, as shown in Table 6 and Table 7. Therefore, compared with the single component, the adsorption of each component of shale oil has decreased to varying degrees. The adsorption ratio of multi-component shale oil is higher than that when the light component and the medium component exist alone, but lower than that when the medium component and the heavy component exist alone, as shown in Table 6 and Table 7 and Figure 10. As shown in Figure 9, the bulk density of the medium component and the light component is basically the same, and the density of the dissolved gas and the medium component is also almost the same, and the former is significantly higher than the latter. The ratio of the first adsorption peak density of the medium component and the heavy component to the free state density has increased significantly compared with the single component, reflecting that the long-chain molecules are in a dominant position in the competitive adsorption of the components, while the dissolved gas and light components have decreased. The detailed adsorption layer parameters are shown in Table 7.

**Figure 11:**
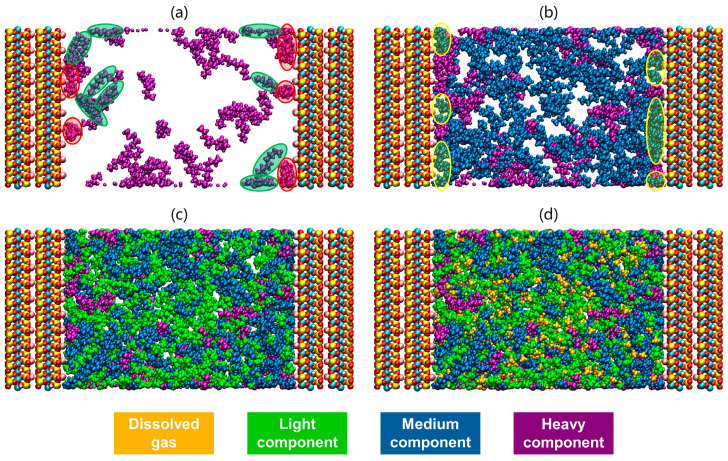
Adsorption states of different shale oil components. (**a**) Heavy component (**b**) Adding display of medium component (**c**) Adding display of light component (**d**) Adding display of dissolved gas.

Among the four shale oil components, the heavy component has the strongest interaction force with the illite wall and is more inclined to be preferentially adsorbed by the wall. However, due to the smallest molar fraction of the heavy component, it cannot occupy too many adsorption sites of the wall. The red elliptical area in Figure 11a is the heavy component adsorption site, which only occupies a small part of the illite wall. The heavy component has the longest carbon chain. Due to the influence of other components in the pore, the orientation of many heavy component molecules near the adsorption layer is not parallel to the wall, and some are even nearly perpendicular to the wall, as shown in the green elliptical area in Figure 11a. These molecules contribute little to the peak of the first adsorption layer of the heavy component, resulting in a very small peak density of the first adsorption layer of the heavy component, and only one obvious adsorption layer is formed. Compared with the adsorption layer when the heavy component exists alone in the pore, the adsorption of the heavy component is greatly weakened when there are multiple components, as shown in Figure 10d and Figure 11a.

The interaction force between the medium component and the illite wall is second only to the heavy component, and the carbon chain of the medium component is much shorter than that of the heavy component, so it will preferentially occupy the remaining adsorption sites after a large number of heavy components are adsorbed. Due to the short carbon chain, the molecular orientation is less affected by other component molecules during the adsorption process. The number of molecules in the medium component that are oriented parallel to the wall is much larger than that of the heavy component, as shown in the yellow elliptical area in Figure 11b. These molecules contribute greatly to the peak density of the first adsorption peak of the medium component, which is also the microscopic reason why the first adsorption peak density of octane is the largest and the adsorption phase accounts for the highest proportion.

The heavy and medium components with greater interaction forces with the illite wall occupy most of the adsorption sites, and the light components do not have enough solid–liquid interaction forces and adsorption sites to form adsorption layers and adsorption peaks. However, due to the small molecules of the light components and the large molar fraction, the small adsorption space remaining near the wall is still occupied by a large number of light components, forming an adsorption layer.

The interaction force between the dissolved gas and the illite wall is further reduced. Most of the space near the illite wall has been occupied by other components, leaving only a small amount of smaller adsorption space that can be adsorbed by the dissolved gas. Therefore, the first adsorption peak of the dissolved gas is the smallest among all components, and the adsorption amount accounts for the smallest proportion.

It can be seen from Table 6 that when shale oil exists independently as a single component, the proportion of shale oil adsorption increases with the increase in carbon number of each component and the enhancement of illite solid–liquid interaction. However, for multi-component shale oil, according to the mechanism analysis of the adsorption characteristics of different components, the proportion of adsorption not only increases with the increase in carbon number of each component, but also decreases with the decrease in mole fraction. If a component has a relatively high carbon number but a very low mole fraction, the proportion of adsorption may be very low and most of the fluid is in a free state. This also explains why some of the produced shale oil contains considerable heavy components, and due to the decrease in the temperature and pressure of shale oil during oil production, a large amount of light components in the produced oil are lost, resulting in higher proportion of heavy components. As shown in Table 7.

### 4.4. Effects of Temperature and Pressure on Multi-Component Shale Oil Adsorption

**Figure 12:**
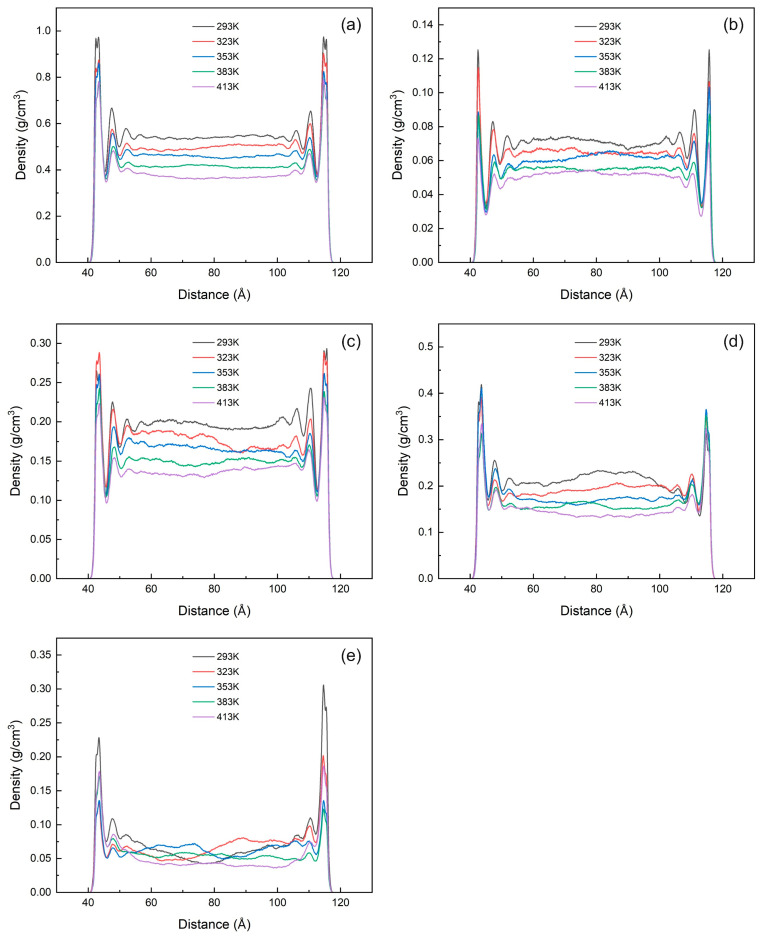
Mass density distribution of shale oil and its components at the same pressure (20 MPa) and different temperatures (293 K, 323 K, 353 K, 383 K, 413 K). (**a**) Shale oil (**b**) Dissolved gas (**c**) Light component (**d**) Medium component (**e**) Heavy component.

We investigated the effects of temperature and pressure on multi-component shale oil adsorption by simulating the adsorption in the illite nanopores both under varying temperatures (293 K, 323 K, 353 K, 383 K, 413 K) at constant pressure (20 MPa) and varying pressures (10 MPa, 20 MPa, 30 MPa, 40 MPa, 50 MPa) at constant temperature (353 K), as shown in Figures 12 and 15.

**Figure 13:**
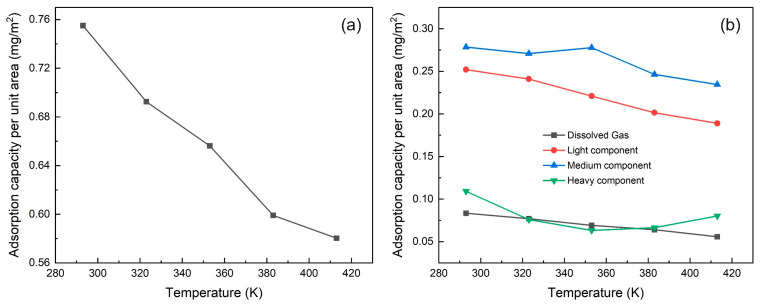
Adsorption of shale oil and its components at different temperatures (293 K, 323 K, 353 K, 383 K, 413 K) under 20 MPa (**a**) Shale oil (**b**) Shale oil components (dissolved gas, light component, medium component, heavy component).

When the temperature rises, the kinetic energy of shale oil increases at high temperature, and the adsorption capacity of the illite wall for shale oil weakens. Although the thickness of single-layer adsorption remains almost unchanged, the peak density of the adsorption layer continues to decrease. A large amount of adsorbed shale oil is desorbed to a free state, and the adsorption amount also decreases from 0.76 mg/m^2^ at 293 K to 0.58 mg/m^2^ at 413 K, as shown in Figure 12a and Figure 13a. It can be seen from Figure 12b–e that except for the heavy component, other components form three adsorption layers, and the adsorption peak increases with temperature. This is consistent with the changing trend of shale oil. The heavy component forms three adsorption layers at low temperature, which gradually decreases to two as the temperature rises. The free density of each component decreases with increasing temperature (this is consistent with the research conclusion of Xu et al. [59], but different from the research conclusion of Xue et al. [6]). When the molecular weight increases, the fluctuation of the free density becomes larger, the adsorption layers on both sides of the illite wall are no longer symmetrically distributed, and the free density of the heavy component has no obvious trend of changing with temperature. This is because the heavy component has a large molecular weight, a small mole fraction, and is difficult to distribute evenly.

The simulation results of Figure 13b show that as the temperature increases, the adsorption per unit area of the light and medium components of shale oil continues to decrease, the dissolved gas decreases slightly, and the adsorption of the heavy component decreases first and then increases, with little change in adsorption. This is because when the temperature increases, a large number of light and medium components are preferentially desorbed, and the heavy components with higher molecular weight are still not desorbed, so they have relatively stronger adsorption competitiveness [26] and can occupy more adsorption space, resulting in an increase in the adsorption of the heavy component. These mechanistic conclusions indicate that the light and medium components are the main contributors to thermal recovery, and the heavy components are still accumulated in the shale nanopores, which is consistent with the research conclusions of Yang et al. [60] and Zhang et al. [61].

In this work, in order to further clarify the effect of temperature on illite pore shale oil, we use the mean square displacement (MSD) method to calculate the self-diffusion coefficient of shale oil and its components. It relies on the Einstein relation stating that MSD is linearly related to diffusion time [62],

(6) $$D=\frac{1}{6N}\underset{t\to \infty }{\lim}\frac{d}{dt}\sum_{i-1}^{N}<{\left[r_{i}\left(t\right)-r_{i}\left(0\right)\right]}^{2}>$$

where *N* presents the amount of target molecules in the system, $r_{i}\left(0\right)$ is the position of molecule *i* at the initial time. $r_{i}\left(t\right)$ is the position of molecule *i* at time *t*.

**Figure 14:**
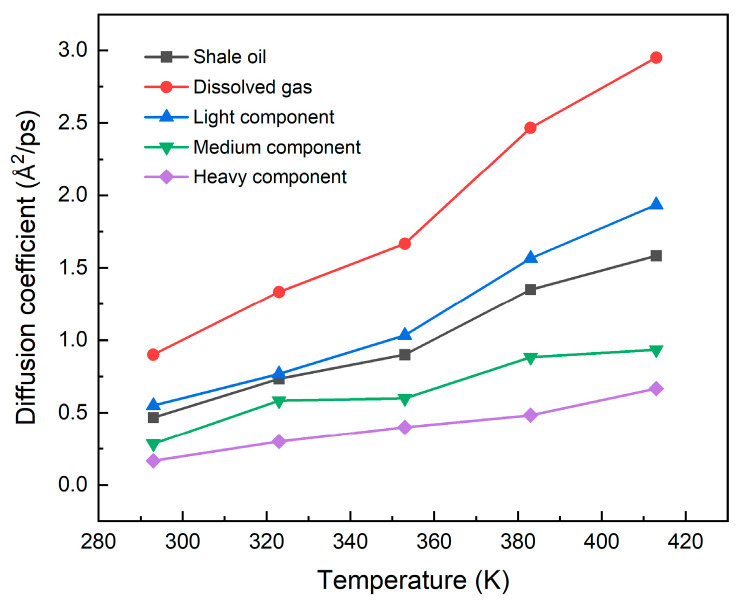
Self-diffusion coefficients of shale oil and its components at different temperatures (293 K, 323 K, 353 K, 383 K, 413 K) at 20 MPa.

The calculation results of the self-diffusion coefficient are shown in Figure 14. The results show that when the temperature rises, the smaller the molecular weight of each component of shale oil and the lower the carbon number, the more significant the increase in the self-diffusion coefficient, and the easier it is for the adsorbed state to desorb to the free state. The self-diffusion change trend of shale oil is consistent with the change trend of light and medium components, which also shows that light and medium components are the main contributors to the thermal recovery of shale oil. Although the self-diffusion coefficient of the heavy component has increased, the increase is relatively small, and the diffusion coefficient is still very small, so its contribution to the diffusion of shale oil is minimal.

**Figure 15:**
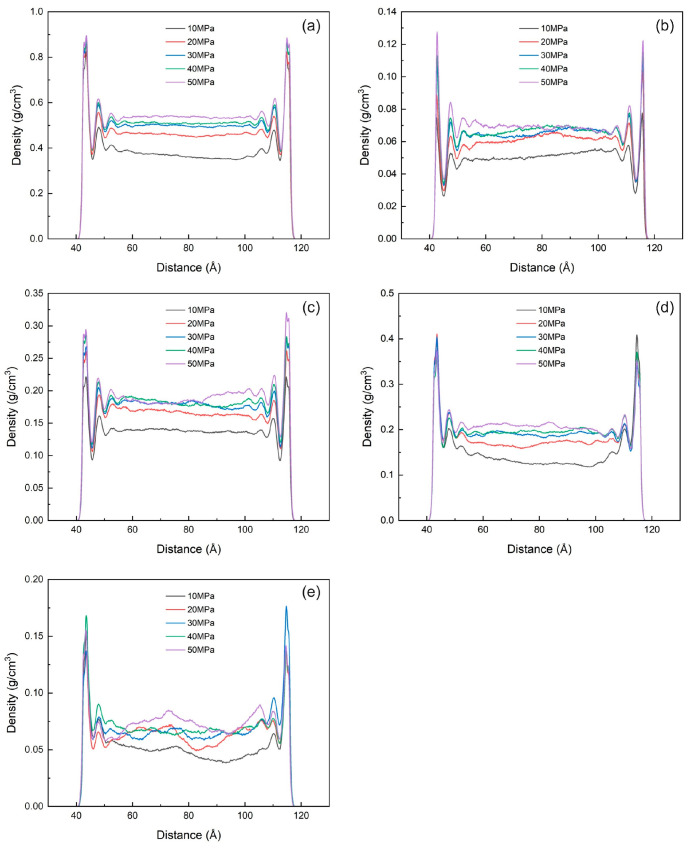
Mass density distribution of shale oil and its components at different pressures (10 MPa, 20 MPa, 30 MPa, 40 MPa, 50 MPa) at 353 K (**a**) shale oil (**b**) dissolved gas (**c**) light component (**d**) medium component (**e**) heavy component.

**Figure 16:**
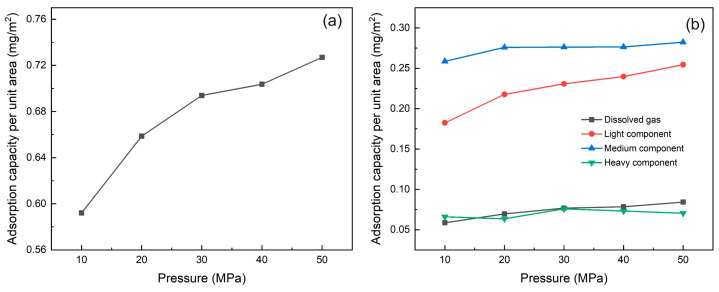
The adsorption amount of shale oil and its components at different pressures (10 MPa, 20 MPa, 30 MPa, 40 MPa, 50 MPa) at 353 K (**a**) Shale oil (**b**) Shale oil components (dissolved gas, light component, medium component, heavy component).

The mass density distribution of shale oil after reaching adsorption equilibrium at different pressures is shown in Figure 15a. When the reservoir pressure increases, the adsorption of shale oil in illite pores is promoted. The heavy components near the wall form two adsorption layers, and the other three components form three adsorption layers. The thickness of the single adsorption layer remains almost unchanged. The peak density of the first adsorption layer remains unchanged or increases slightly with the increase in pressure. The peak density of other adsorption layers and the density of free shale oil also increase, but the increase rate continues to decrease. The density of the free state of the heavy component of shale oil fluctuates greatly, and there is no obvious trend of change with pressure. This is because the molecular weight of the heavy component is large, and the spatial change of molecular distribution caused by pressure change has limited effect on it. The adsorption amount of shale oil under different pressures is shown in Figure 16a.

As shown in Figure 16, when the pressure increases, the adsorption amount of shale oil increases accordingly. The adsorbed components of shale oil are mainly the light component and the medium component. The increase in adsorption amount is mainly contributed by the medium components. The adsorption amount of dissolved gas and light components only increases slightly, and the adsorption amount of heavy components hardly changes. The effect of pressure increase on adsorption amount of the heavy component can be ignored, which is consistent with the research conclusions of Xu et al. [59].

**Figure 17:**
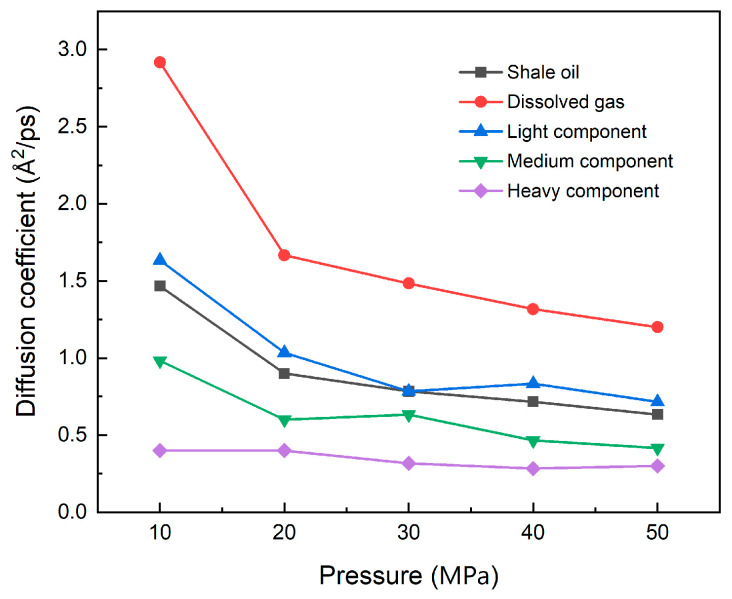
Self-diffusion coefficients of shale oil and its components at different pressures (10 MPa, 20 MPa, 30 MPa, 40 MPa, 50 MPa) at 353 K.

Figure 17 shows the diffusion coefficients of shale oil and its different components. As can be seen from the figure, when the pressure increases, the space that shale oil molecules can enter continues to decrease, which inhibits the migration of shale oil in illite nanopores and reduces the self-diffusion ability. The smaller the molecular weight of the shale oil component, the greater the pressure effect, resulting in a sharp decrease in the diffusion coefficient in the initial stage of pressure increase, as shown in Figure 17. When the increase in pressure causes the migration space of the molecules to be compressed to a great extent, the compression effect on the molecular migration space becomes smaller if the pressure increases further, and the rate of decrease in the diffusion coefficient also slows down. The rate of decrease in different shale oil components gradually tends to be consistent. In the process of pressure increasing, the change in the molecular migration space has little effect on diffusion movement of heavy components because of their long carbon chain and large molecular weight, so the diffusion coefficient remains stable at a low level.

### 4.5. Effect of Pore Width on Shale Oil Adsorption in Pores

**Figure 18:**
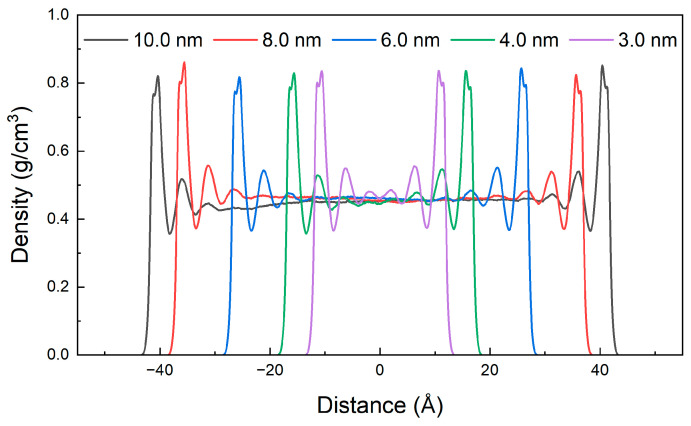
Mass density distribution of shale oil with different pore widths at 353 K and 20 MPa.

**Figure 19:**
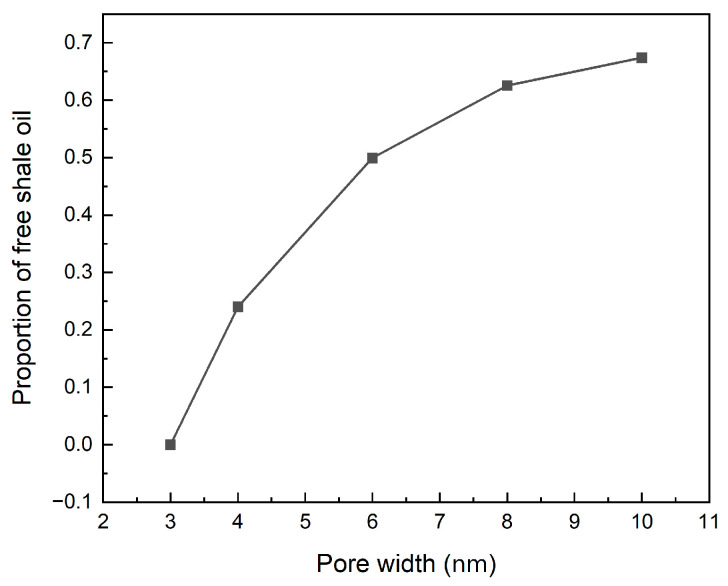
Percentage of free shale oil at different pore widths at 353 K and 20 MPa.

In this work, the illite nanopore models featuring a range of pore diameters, including 3.0 nm, 4.0 nm, 6.0 nm, 8.0 nm and 10.0 nm, were constructed to reveal the effect of pore diameters on the occurrence of shale oil. MDSs were then conducted to analyze the shale oil’s adsorption and behaviors within these nanopores. As seen from the results displayed in Figure 18 by the mass density curve, the shale oil near the two side walls of illite is distributed symmetrically along the center of the pore. When the pore width is 3.0 nm, due to the small distance between the illite wall and the shale oil molecules, all the shale oil molecules in the pore are completely in an adsorption state because of the solid–liquid attraction of the wall, and three adsorption layers are formed near the illite wall on each side; when the pore increases to 4.0 nm, the solid–liquid interaction in the central area of the pore is very weak, and free shale oil has appeared. Therefore, 3.0 nm–4.0 nm becomes the critical value of the pore width for whether shale oil exists in a free state at 353 K and 20 MPa; when the pore width continues to increase, the number of shale oil adsorption layers no longer changes, and the peak density of each adsorption layer remains almost unchanged, and the amount of free shale oil continues to increase. The stability of adsorption indicates that the adsorption of shale oil by the illite wall has reached saturation. As the pore width increases further, the proportion of adsorbed shale oil decreases, while the proportion of free shale oil increases. Figure 19 shows the proportion of free shale oil at different pore widths. It also shows that when the reserves of shale oil reservoirs are constant, increasing the pore width through hydraulic fracturing, thereby improving reservoir physical properties, is an effective method to increase the proportion of free shale oil and improve recovery.

## 5. Conclusions

In this work, the multi-component shale oil model with light hydrocarbon correction, being closer to the real shale oil in reservoir environments, was constructed for molecular dynamics simulation, and the adsorption behavior of multi-component shale oil in illite nanopores was studied. The effects of adsorption characteristics and different reservoir environments on adsorption were also discussed. The main conclusions are as follows:

(1) Under reservoir conditions, multi-component shale oil has multi-layer adsorption, and the thickness of each adsorption layer is about 0.46 nm. The microscopic solid–liquid interactions between each component and the illite wall are as follows from large to small: heavy component, medium component, light component, dissolved gas. There is a competitive adsorption between different components. The heavy component is preferentially adsorbed on the wall, but due to the small molar fraction and the long molecular carbon chain, it only occupies a small part of the adsorption sites, the peak density of the adsorption layer is small, and most of the molecules are still in free state; the adsorption amount of the medium component accounts for the largest proportion; the light component and dissolved gas are mainly enriched in the free zone. Therefore, the adsorption ratios of different components increase with the increase in molecular weight and decreases with the decrease in molar fraction. That is to say, the adsorption ratio is affected by both molecular size and mole fraction. It is also one of the reasons why a considerable amount of heavy components appears in some produced shale oil.

(2) When the temperature of reservoir increases, the shale oil adsorption amount and the peak density of the adsorption layer decrease, but the thickness of the single layer of the adsorption remains unchanged. The light and medium components desorb significantly, and the heavy components still accumulate in the nanopores and are less affected by temperature changes. The increase in reservoir pressure inhibits the migration of shale oil in the nanopores, the self-diffusion capacity decreases, and the lighter components decrease more. However, the adsorption amount is less affected by pressure. The adsorption amount of light components increases a little, and that of other components changes slightly. The heavy components remain stable at a low level. Therefore, the increase in pressure does not significantly increase the adsorption and enrichment of shale oil, but rather inhibits the diffusion of shale oil.

(3) When the pore width increases, the adsorption of shale oil gradually reaches saturation, and the adsorption state remains almost unchanged. At this time, the pore width (3.0 nm–4.0 nm) is the critical width for the existence of free shale oil. As the pore width decreases, shale oil is completely adsorbed in the pores. When the width increases further, free shale oil begins to appear and continues to increase. As a result, the proportion of adsorbed shale oil continues to decrease, while the proportion of free shale oil continues to increase. Therefore, increasing the pore width through hydraulic fracturing can effectively increase the proportion of free shale oil and improve shale oil recovery.

## Figures and Tables

**Table 1:** Illite unit cell parameters.

Atom	x	y	z
K	0	0.5	0.5
Al	0.5	0.1667	0
Si	0.4189	0.3279	0.2690
O1	0.3474	0.3086	0.1060
O2	0.5013	0.5	0.3123
O3	0.6697	0.2229	0.3350
OH	0.4188	0	0.0998

**Table 2:** Composition of the multi-component shale oil model.

Component of Shale Oil Model	Representative Substance	Molar Mass (g/mol)	Mass Fraction (%)	Mole Fraction (%)
Dissolved gas	CH_4_	16.0425	12.72	39.6
Light Component	C_3_H_8_	44.0956	35.48	40.2
Medium Component	C_8_H_18_	114.2258	38.77	17.0
Heavy Component	C_14_H_30_	198.388	13.03	3.2

**Table 3:** Non-bond parameters for illite from the ClayFF force field.

Species	Symbol	Charge (e)	Mass (g/mol)	ε (kcal/mol^−1^)	σ (Å)
tetrahedral silicon	st	2.1	28.0855	1.8405 × 10^−6^	3.302
tetrahedral aluminum	at	1.575	26.9815	1.8405 × 10^−6^	3.302
octahedral aluminum	ao	1.575	26.9815	1.3298 × 10^−6^	4.2712
hydroxyl hydrogen	ho	0.425	1.0079	/	/
hydroxyl oxygen	oh	−0.95	15.9994	0.1554	3.1655
bridging oxygen	ob	−1.05	15.9994	0.1554	3.1655
bridging oxygen with tetrahedral substitution	obts	−1.1688	15.9994	0.1554	3.1655
aqueous potassium ion	K	1	39.0983	0.1	3.334

**Table 4:** Non-bond parameters for n-alkane from the OPLS–AA and the L–OPLS–AA force fields.

Species	Symbol	Charge (e)	Mass (g/mol)	ε (kcal/mol)	σ (Å)
methane carbon	c	−0.24	12.0108	0.066	3.5
methyl carbon (propane)	c3	−0.18	12.0108	0.066	3.5
methylene carbon (propane)	c2	−0.12	12.0108	0.066	3.5
alkane hydrogen (methane, propane)	h	0.06	1.0079	0.03	2.5
methyl carbon (octane, tetradecane)	c3	−0.222	12.0108	0.066	3.5
methylene carbon (octane, tetradecane)	c2	−0.148	12.0108	0.066	3.5
alkane hydrogen (octane, tetradecane)	h	0.074	1.0079	0.03	2.5

**Table 5:** Bond parameters for n-alkane from the OPLS–AA force field.

bond stretching	symbol	$K_{r}$ (kcal/mol/Å^2^)		$r_{0}$ (Å)	
	oh-ho	554.1349		1	
	c-c	268		1.529	
	h-c	340		1.09	
bond angle bending	symbol	$K_{\theta }$ (kcal/mol/rad^2^)		$\theta_{0}$ (°)	
	h-c-h	33		107.8	
	h-c-c	37.5		110.7	
	c-c-c	58.35		112.7	
dihedral angle torsion	symbol	$c_{1}$ (kcal/mol)	$c_{2}$ (kcal/mol)	$c_{3}$ (kcal/mol)	$c_{4}$ (kcal/mol)
	h-c-c-h	0	0	0.3	0
	h-c-c-c	0	0	0.3	0
	c-c-c-c	0.645	−0.214	0.178	0

**Table 6:** Adsorption layer parameters of different other representative components.

Representative Component	Layers	Average Thickness of Layers (nm)	Peak Densities of Layers (g/cm^3^)	Free Fluid Density (g/cm^3^)	Ratio of the 1st Peak Density to the Free Density	Adsorption Capacity per Unit Area (mg/m^2^)	Adsorption Percentage (%)
Methane	2	0.38	0.298	0.117	2.5	0.11	22.9
Propane	3	0.47	0.784	0.464	1.7	0.66	37.2
			0.549				
			0.488				
Octane	4	0.46	1.173	0.687	1.7	1.27	48.6
			0.845				
			0.750				
			0.710				
Tetradecane	4	0.47	1.324	0.748	1.8	1.41	49.5
			0.981				
			0.833				
			0.782				

**Table 7:** Adsorption layer parameters of shale oil.

Shale Oil Component	Layers	Molar Fraction (%)	Average Thickness of Layers (nm)	The 1st Peak Densities of Layers (g/cm^3^)	Free Fluid Density (g/cm^3^)	Ratio of the 1st Peak Density to the Free Density	Adsorption Percentage (%)
Shale oil	3	1	0.46	0.862	0.459	1.9	38.3
Dissolved gas	2	39.6	0.46	0.089	0.062	1.44	18.9
Light component	3	40.2	0.46	0.260	0.167	1.56	23.7
Medium component	3	17.0	0.48	0.411	0.169	2.43	31.4
Heavy component	1	3.2	0.45	0.136	0.063	2.16	25.1

## Data Availability

The data supporting the findings of this study can be obtained from the corresponding author upon reasonable request.
